# Serum metabolic characteristics of interstitial lung disease: a potential link between endocrine aging and gut-derived metabolites

**DOI:** 10.3389/fmed.2026.1891114

**Published:** 2026-08-14

**Authors:** Lu Liu, Xinyi Wang, Jinling Xiao, Yandong Li, Shihuan Yu

**Affiliations:** 1 The First Affiliated Hospital of Harbin Medical University, Harbin, China; 2 The Second Affiliated Hospital of Heilongjiang University of Traditional Chinese Medicine, Harbin, China

**Keywords:** biomarkers, endocrine aging, interstitial lung disease, lipid remodeling, untargeted metabolomics

## Abstract

**Objective:**

To analyze differences in serum metabolic profiles between patients with interstitial lung disease (ILD) and healthy controls in northern China using untargeted metabolomics techniques, to identify differentially expressed metabolites and key dysregulated pathways, and to identify potential metabolic biomarkers.

**Methods:**

Thirty patients with interstitial lung disease (ILD) and 30 healthy controls were enrolled. Untargeted metabolomics analysis was performed using LC-MS/MS in both positive and negative ion modes. Differentially expressed metabolites were identified using the criteria VIP > 1.0, FC > 1.2 or FC < 0.833 and *P*-value < 0.05. Pathway enrichment analysis was conducted using the KEGG database, and diagnostic performance was evaluated using ROC curves.

**Results:**

The global metabolic profile showed significant separation between the ILD group and the control group. Pathway enrichment analysis revealed significant dysregulation in lipid metabolism (particularly linoleic acid metabolism), amino acid biosynthesis, and steroid hormone metabolism. GSEA analysis further confirmed the suppression of overall metabolic activity and the specific enrichment of tryptophan metabolism. Among the differentially expressed metabolites, DG(15:0/18:2(9Z,12Z)/0:0) (AUC = 0.914), Medroxalol (AUC = 0.912), estriol 3-sulfate (AUC = 0.890) and DHEA-S (AUC = 0.877) demonstrated high diagnostic value.

**Conclusion:**

Untargeted metabolomics has revealed significant systemic metabolic dysregulation in ILD. The biomarkers and “metabolic-immune-endocrine” interaction patterns identified offer potential leads for early diagnosis and targeted treatment, which require validation in larger cohorts.

## 1 Introduction

Interstitial lung disease (ILD) is a heterogeneous group of conditions characterized primarily by inflammation and fibrosis of the pulmonary interstitium. It comprises over 200 subtypes with diverse etiologies, encompassing idiopathic, autoimmune and environmentally-related forms, among others (1). Although the pathological mechanisms and clinical courses of the various subtypes differ significantly, they share the common features of progressive decline in lung function and poor prognosis. Among these, idiopathic pulmonary fibrosis (IPF), as the most common subtype of ILD, has a median survival of only 3–5 years in untreated patients, which is even lower than that of many malignant tumors (2). Certain specific subtypes, such as anti-MDA5 antibody-positive ILD, progress even more rapidly, leading to respiratory failure and death within a matter of weeks, highlighting the high lethality of ILD (3).

Currently, the clinical management of ILD still faces multiple challenges. Firstly, studies indicate that the median time from symptom onset to diagnosis ranges from 13 months to 2.1 years, with as many as 98% of patients having been misdiagnosed with common conditions such as asthma or chronic obstructive pulmonary disease (COPD) prior to confirmation (4). Although high-resolution CT is a core diagnostic tool for ILD, manual interpretation is subject to limitations such as high subjectivity and difficulty in identifying early signs; whilst pathological biopsy is the gold standard, its invasive nature restricts its widespread use. More critically, there is a general lack of specific serological diagnostic markers for ILD, making accurate diagnosis particularly difficult, especially for seronegative patients. Secondly, the course of ILD is highly variable; some patients may rapidly progress to progressive pulmonary fibrosis, whilst existing prognostic scoring systems have been insufficiently validated in non-IPF subtypes, making it difficult to accurately predict individual disease trajectories. Currently relied-upon monitoring indicators, such as lung function and HRCT, exhibit relatively delayed changes, making it difficult to detect early disease activity. Finally, existing antifibrotic drugs can only delay rather than reverse the decline in lung function, and there is significant inter-patient variability in response. For patients in advanced stages, although lung transplantation is the only effective treatment, donor shortages and high mortality during the waiting period remain insurmountable obstacles. Consequently, there is an urgent clinical need to identify novel biomarkers capable of enabling early diagnosis, accurate prognostic assessment and dynamic monitoring of disease progression, and to gain a deeper understanding of the molecular mechanisms underlying ILD, thereby providing a basis for precision diagnosis and treatment.

In recent years, metabolomics, as a key branch of systems biology, has provided a fresh perspective on the study of disease mechanisms. Compared with genomics and proteomics, which operate upstream of the central dogma, metabolomics is situated at the downstream end of biological regulatory processes; changes in metabolites are most closely aligned with the organism's actual phenotype, enabling a more sensitive amplification of pathophysiological alterations arising from gene-environment interactions. The fibrotic progression of ILD is accompanied by significant oxidative stress, metabolic reprogramming of myofibroblasts, and extracellular matrix deposition; these processes all involve disturbances in specific metabolic pathways (5–7). As the body's circulating pool, serum not only reflects local lesions in the lungs but also reveals systemic metabolic disturbances in the context of disease (8).

Research on the metabolomics of ILD has made considerable progress to date. Multiple studies have confirmed that lipid metabolism disorders are one of the core characteristics of fibrotic lung diseases, involving multiple lipid families such as glycerides, glycerophospholipids and sphingolipids; these changes are closely associated with pulmonary surfactant dysfunction and fibroblast activation (9). In recent years, the focus of research has gradually shifted from comparing differences between ILD and healthy individuals to distinguishing disease subtypes and predicting disease progression. For example, Tang et al. (10) conducted a multi-omics integrated analysis of organizing pneumonia (OP) and found that lipid metabolic reprogramming plays a central role in the disease process. This is characterized not by a simple overall increase or decrease in lipid levels, but rather by chain-length-dependent alterations in specific lipid molecules. Further mechanistic studies have shown that C16Cer promotes ferroptosis in macrophages by inhibiting the GSH/SLC3A2/GPX4/FTH1/FTL axis, and induces macrophage secretion of DPP7 and FABP4, thereby driving epithelial cell fibrosis. Furthermore, the roles of energy metabolism reprogramming and amino acid metabolism abnormalities in the pathogenesis of ILD are increasingly being elucidated. Existing studies indicate that TGF-β-induced myofibroblast differentiation depends on alanine synthesis and transport, whilst mitochondrial dysfunction in alveolar macrophages has also been demonstrated to be associated with the ILD state (11, 12). However, existing research still has certain limitations; most current metabolomics studies focus on specific subtypes such as IPF or concentrate on single metabolite categories such as lipids, and there is still a lack of systematic characterization of the panoramic differences in serum metabolic profiles between the entire heterogeneous group of ILD patients and healthy individuals.

As a heterogeneous group of diseases, the interaction between local pulmonary lesions and systemic metabolic disorders in ILD remains to be elucidated in depth. Therefore, this study employs untargeted metabolomics techniques to conduct a comprehensive analysis of serum samples from patients with interstitial lung disease and healthy controls. The aim is to systematically characterize the differences in their metabolic profiles, identify potential diagnostic biomarkers, and explore the mechanisms of metabolic dysregulation associated with the onset and progression of ILD through metabolic pathway analysis, thereby providing new insights for the early identification and mechanistic research of this disease.

## 2 Methods

### 2.1 Study population

This study included a total of 60 participants: 30 in the ILD group, comprising patients diagnosed with ILD and admitted to the First Affiliated Hospital of Harbin Medical University for inpatient treatment; and 30 in the healthy control group (HC group), comprising healthy subjects who underwent medical examinations at the same hospital during the same period. This study was approved by the Ethics Committee of the First Affiliated Hospital of Harbin Medical University (Ethics No.: 2026120), and the research process strictly adhered to the ethical requirements of the latest revised edition of the Declaration of Helsinki. Serum samples from all participants were collected between 7 April 2026 and 27 May 2026, with sampling conducted during the same season for both the case group and the control group to minimize the potential influence of environmental factors such as season and temperature on serum metabolomic profiles.

Inclusion criteria for the ILD group: (1) age ≥ 40 years; (2) meeting the diagnostic criteria for ILD, with typical interstitial changes confirmed by HRCT imaging; (3) voluntary participation and signing of an informed consent form. Exclusion criteria: (1) concurrent severe infection, systemic inflammatory disease or malignant tumor; (2) severe hepatic or renal insufficiency; (3) pregnant or breastfeeding women.

Inclusion criteria for the healthy control group: (1) Age ≥ 40 years; (2) No history of respiratory diseases and no abnormalities on chest imaging; (3) No severe cardiac, hepatic, renal or metabolic diseases; (4) Voluntary participation and signing of an informed consent form.

### 2.2 Collection and processing of serum samples

All study participants had 5 mL of peripheral venous blood collected in the early morning whilst fasting. The blood was placed in anticoagulant-free vacuum blood collection tubes. After standing at room temperature for 30 min, the tubes were centrifuged at 1,500 rpm at 4 °C for 15 min. The supernatant serum was collected, aliquoted into sterile Eppendorf tubes, and rapidly stored in a −80 °C ultra-low temperature freezer to avoid repeated freeze-thaw cycles. Metabolomic analysis was performed on all samples within 1 month of collection. Prior to analysis, samples were slowly thawed in a 4 °C refrigerator. Following thawing, they were centrifuged again (2,000 rpm, 4 °C, 10 min), and the supernatant was collected for subsequent analysis.

### 2.3 Untargeted metabolomics

#### 2.3.1 Metabolite extraction

Place 100 μL of sample into an EP tube, add 400 μL of an 80% methanol-water solution, vortex, then leave to stand on an ice bath for 5 min, and centrifuge at 15,000 g and 4 °C for 20 min. Collect the supernatant, add 204 μL of mass spectrometry-grade water (to give a final methanol concentration of 53 per cent), and centrifuge again at 15,000 g and 4 °C for 20 min. Collect the supernatant and set it aside for subsequent LC-MS analysis (13).

#### 2.3.2 Instrument parameters

Chromatographic conditions: Hypersil Gold column (C18) was used, with a column temperature of 40 °C and a flow rate of 0.2 mL/min; mobile phase A consisted of ultrapure water containing 0.1% formic acid, and mobile phase B consisted of methanol. Mass spectrometry conditions: scan range m/z 100–1,500; An ESI ion source was employed with a spray voltage of 3.5 kV, sheath gas flow rate of 35 psi, auxiliary gas flow rate of 10 L/min, ion transfer tube temperature of 320 °C, ion source RF level of 60, and auxiliary gas heater temperature of 350 °C. Data were acquired in both positive and negative ion modes; MS/MS secondary scans utilized data-dependent scanning (data-dependent scans).

### 2.4 Data processing and quality control

Raw data was converted to mzXML format using ProteoWizard software (ProteoWizard Software Foundation, San Francisco, CA, USA), followed by peak extraction, peak quantification and inter-sample peak alignment using XCMS software (with retention time and mass-to-charge ratio as core parameters for correcting peak matching discrepancies). Metabolite identification was performed using R version 3.4.3 with a mass tolerance of 10 ppm (14), combining adduct information with the Novogene-developed in-house high-resolution MS/MS database (NovoMetDB). During the data pre-processing stage, metabolites with a missing value rate exceeding 50% were first excluded; the remaining missing values were imputed using the K-nearest neighbors (KNN) algorithm in Python 3.5.0 (Python Software Foundation, Wilmington, DE, USA) (15), followed by background ion subtraction using a blank sample; Subsequently, the raw quantitative results were standardized using R version 3.4.3 (R Foundation for Statistical Computing, Vienna, Austria), and compounds with a coefficient of variation (CV) in relative peak area exceeding 30% in the QC samples were filtered out (16), ultimately yielding standardized relative peak area data for the metabolites. All data processing workflows were executed on the Linux operating system (CentOS version 6.6; The CentOS Project, USA).

### 2.5 Statistical analysis

#### 2.5.1 Global metabolomic profiling analysis

Unsupervised principal component analysis (PCA) was performed on the pre-processed metabolomic data to examine the overall metabolic differences between the ILD group and the HC group, as well as the clustering of QC samples. Furthermore, a supervised orthogonal partial least squares discriminant analysis (OPLS-DA) model was constructed, and its validity was verified via 200-fold cross-validation to avoid overfitting.

#### 2.5.2 Screening of differentially expressed metabolites

Raw mass spectrometry data underwent pre-processing using metaX software (MetaX Software, Shenzhen, Guangdong, China) for peak extraction, alignment and normalization, followed by centering and scaling to eliminate differences in units. Orthogonal partial least squares discriminant analysis (OPLS-DA) was utilized to construct a scoring plot to visually illustrate the separation trends between groups and eliminate noise interference; simultaneously, the variable importance in projection (VIP) values for each metabolite were calculated based on a partial least squares discriminant analysis (PLS-DA) model. Finally, differentially expressed metabolites were rigorously screened using a combination of multiple and univariate statistical criteria, with the screening thresholds set as follows: VIP > 1.0, fold change (FC) > 1.2 or FC < 0.833, and *P* < 0.05.The significantly differentially expressed metabolites identified were subjected to functional annotation and metabolic pathway enrichment analysis using the KEGG (https://www.genome.jp/kegg/pathway.html), HMDB (https://hmdb.ca/metabolites) and LIPIDMaps (http://www.lipidmaps.org/) databases to determine their chemical names, structures and associated biological pathways.

#### 2.5.3 Enrichment analysis of differentially expressed metabolite pathways

The screened significantly differentially expressed metabolites were imported into the MetaboAnalyst 6.0 database, and metabolic pathway enrichment analysis was conducted in conjunction with the KEGG database. The enrichment criterion was defined as x/n > y/N (where x is the number of differentially expressed metabolites annotated to a specific pathway, n is the total number of differentially expressed metabolites, y is the total number of metabolites in that pathway within the database, and N is the total number of annotated metabolites in the database), with *P* < 0.05, to identify core metabolic dysregulation pathways associated with ILD. The ggplot2 package was used to plot a bubble plot to visualize the enrichment results.

#### 2.5.4 Analysis of diagnostic performance of potential metabolic biomarkers

To identify potential metabolic biomarkers for ILD, this study utilized MetGSEA enrichment analysis to extract metabolites with “CORE ENRICHMENT = Yes” from the core dysregulated pathways as candidate biomarkers. ROC curves were used to evaluate the discriminatory performance of candidate biomarkers and combinations for ILD, with AUC, sensitivity and specificity as core indicators. An AUC > 0.7 was considered a reference threshold indicating potential diagnostic utility; the results reflect the strength of association rather than definitive diagnostic performance.

## 3 Results

### 3.1 Descriptive analysis

A total of 60 participants were enrolled in this study, comprising 30 patients with Interstitial Lung Disease (ILD) and 30 healthy controls. The baseline demographic and clinical characteristics are summarized in Table 1.

**Table 1:** Baseline characteristics of the study population.

Category	ILD Group, *n* (%)	Control Group, *n* (%)
Total	30 (100.0)	30 (100.0)
Sex		
Male	12 (40.0)	12 (40.0)
Female	18 (60.0)	18 (60.0)
Age, years		
≤ 65	22 (73.3)	25 (83.3)
>65	8 (26.7)	5 (16.7)
BMI		
Underweight	1 (3.3)	0 (0.0)
Normal	11 (36.7)	20 (66.7)
Overweight	14 (46.7)	10 (33.3)
Obese	4 (13.3)	0 (0.0)
WBC, × 10^9^/L		
Normal	25 (83.3)	27 (90.0)
Abnormal	5 (16.7)	3 (10.0)
RBC, × 10^12^/L		
Normal	27 (90.0)	27(90.0)
Abnormal	3 (10.0)	3 (10.0)
Hb, g/L		
Normal	25 (83.3)	26 (86.6)
Abnormal	5 (16.7)	4 (13.3)
PLT, × 10^9^/L		
Normal	28 (93.3)	30 (100.0)
Abnormal	2 (6.7)	0 (0.0)
CRP, mg/L		
Normal	28 (93.3)	30 (100.0)
Elevated	2 (6.7)	0 (0.0)
ALT, U/L		
Normal	29 (96.7)	30 (100.0)
Abnormal	1 (3.3)	0 (0.0)
AST, U/L		
Normal	24 (80.0)	29 (96.7)
Abnormal	6 (20.0)	1 (3.3)
ALB, g/L		
Normal	30 (100.0)	30 (100.0)
Abnormal	0 (0.0)	0 (0.0)
Cr, umol/L		
Normal	29 (96.7)	28 (93.3)
Abnormal	1 (3.3)	2 (6.7)
Pulmonary function severe		
FVC (% pred)		
Normal	15 (50.0)	30 (100.0)
Mild	9 (30.0)	0 (0.0)
Moderate/severe	6 (20.0)	0 (0.0)
DLCO (% pred)		
Normal	7 (23.3)	30 (100.0)
Mild	12 (40.0)	0 (0.0)
Moderate	8 (26.7)	0 (0.0)
Severe	3 (10.0)	0 (0.0)

WBC, white blood cell count; RBC, red blood cell count; Hb, hemoglobin; PLT, platelet count; ALT, alanine aminotransferase; AST, aspartate aminotransferase; ALB, albumin; Cr, creatinine.

Reference intervals were determined by the institutional clinical laboratory: WBC, 3.69–9.16 × 10^9^/L for females and 3.97–9.15 × 10^9^/L for males; RBC, 3.68–5.13 × 10^1^^2^/L for females and 4.09–5.74 × 10^1^^2^/L for males; Hb, 113–151 g/L for females and 131–172 g/L for males; PLT, 98–300.2 × 10^9^/L; CRP, 0–10 mg/L; ALB, 30–50 g/L; AST, 15–46 U/L; ALT, 0–35 U/L for females and 0–50 U/L for males; Cr, 46–92 μmol/L for females and 58–110 μmol/L for males. Severity of lung function impairment was classified based on the percentage of predicted values: FVC: Normal ≥ 80%, Mild 70–79%, Moderate 50–69%, Severe < 50%; DLCO: Normal ≥ 80%, Mild 60–79%, Moderate 40–59%, Severe < 40%.

The study population showed a balanced sex distribution, with 12 males (40.0%) and 18 females (60.0%) in each group. The majority of participants in both the ILD group (73.3%) and the control group (83.3%) were aged ≤ 65 years. Regarding body mass index (BMI), the ILD group exhibited a higher prevalence of overweight and obesity compared to the control group. Specifically, 46.7% of ILD patients were overweight and 13.3% were obese, whereas the control group consisted predominantly of individuals with normal weight (66.7%), with no obese individuals observed.

In terms of laboratory findings, most hematological parameters, including white blood cell count (WBC), red blood cell count (RBC), hemoglobin (Hb), and platelets (PLT), were within normal ranges for the majority of participants in both groups. However, liver function markers showed some variations; abnormal aspartate aminotransferase (AST) levels were observed in 20.0% of the ILD group compared to 3.3% of the controls. Albumin (ALB) levels were normal across all participants.

Pulmonary function tests revealed distinct patterns between the two groups. In the ILD group, Forced Vital Capacity (FVC) was normal in 50.0% of patients, while 30.0% showed mild impairment and 20.0% presented with moderate to severe impairment. In contrast, all subjects in the control group maintained normal FVC values. Similarly, the Diffusing Capacity for Carbon Monoxide (DLCO) was more severely affected in the ILD group: only 23.3% had normal DLCO, while 40.0% showed mild reduction, 26.7% moderate reduction, and 10.0% severe reduction. All controls exhibited normal DLCO levels.

### 3.2 Metabolomics data quality assessment

**Figure 1:**
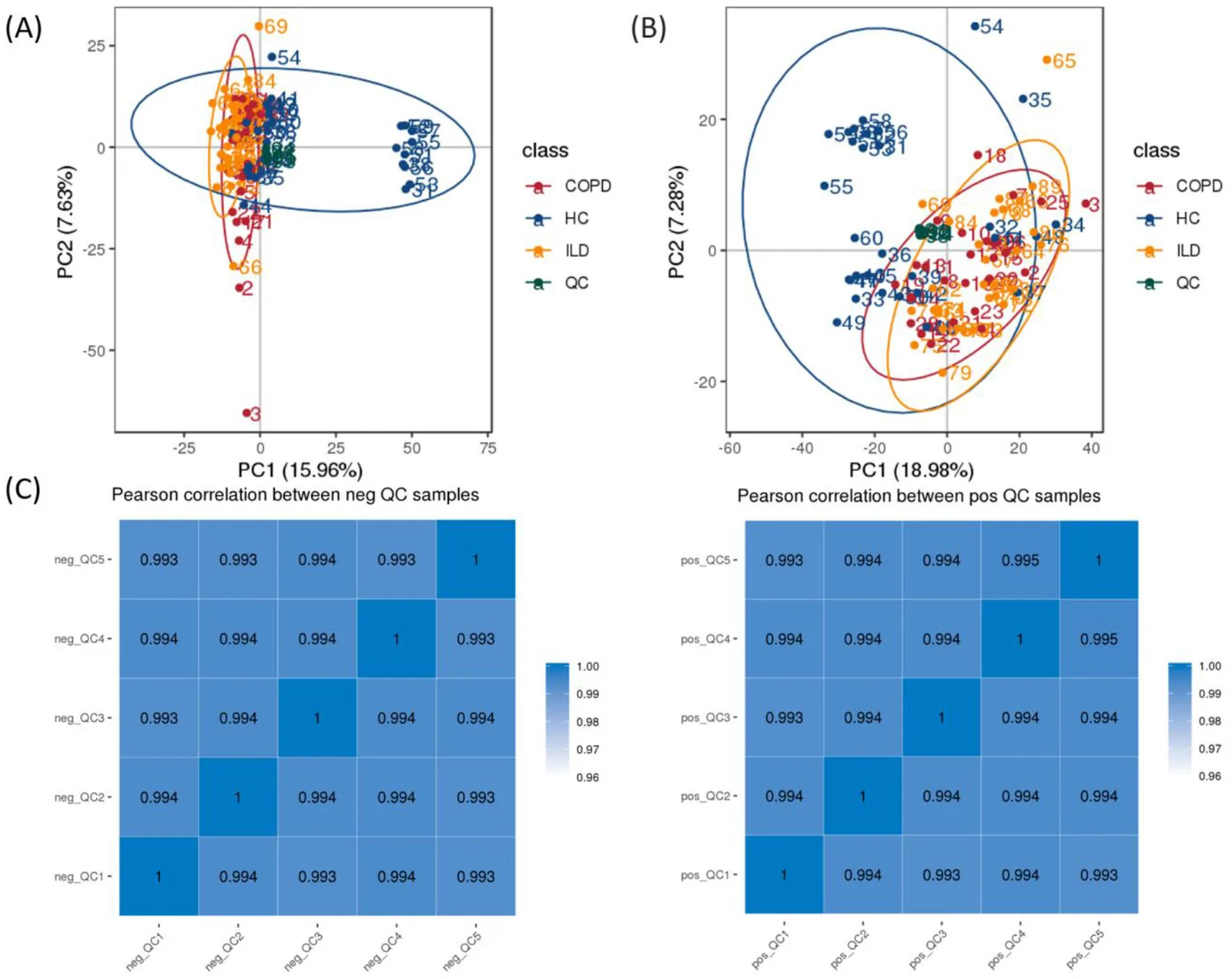
Quality assessment of metabolomics data in positive and negative ion modes. **(A)** PCA plot of experimental samples and QC samples in positive ion mode. The QC samples (green) are tightly clustered, indicating good stability of the detection system. **(B)** PCA plot in negative ion mode, showing consistent results. **(C)** Pearson correlation heatmap of QC samples in both positive and negative ion modes; |r| values are close to 1, further validating the reliability of the data. The COPD group comprises other subgroups from the same experimental run and is not included in the statistical analysis of this study.

In this study, untargeted metabolomics analysis was performed on serum samples. The results of PCA analysis of experimental and QC samples in both positive and negative ion modes showed that all QC samples were closely clustered in the plots, indicating good system stability and excellent experimental reproducibility, as shown in (Figures 1A–C). The Pearson correlation analysis heatmap demonstrates that the correlation coefficients |r| between pairs of QC samples are all close to 1, further validating the reliability of the experimental data and confirming compliance with the quality requirements for untargeted metabolomics data.

### 3.3 Analysis of differences in global metabolic profiles between the ILD group and the HC group

**Figure 2:**
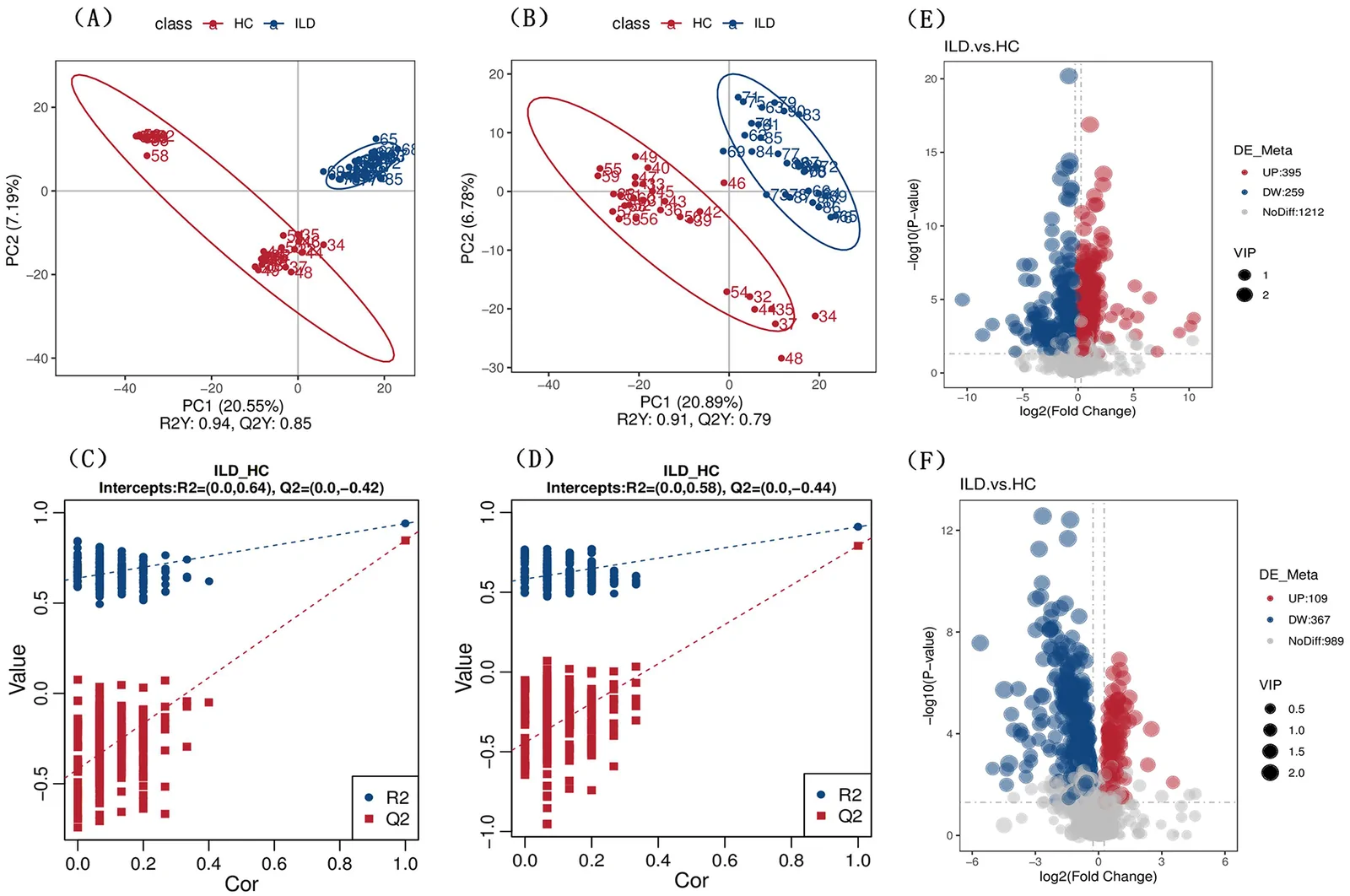
Multivariate statistical analysis of serum metabolomic profiles in the ILD group and the HC group. **(A, B)** OPLS-DA score plots showing the separation between the ILD group (blue) and the HC group (red). The x-axis represents the predictive component, and the y-axis represents the orthogonal component. The ellipses represent the 95% confidence interval. **(C, D)** Permutation tests for the OPLS-DA models, validating model robustness with intercepts *R*^2^ = (0.0, 0.64)/(0.0, 0.58) and *Q*^2^ = (0.0, −0.42)/(0.0, −0.44). **(E, F)** Volcano plots showing differentially expressed metabolites between ILD and HC groups, with VIP values indicated by dot size.

In positive ion mode (Figure 2A), the PLS-DA score plot shows that the ILD group and the HC group are completely separated along the PLS1 principal component axis, with no sample overlap and good intra-group clustering. The model fit parameters *R*^2^Y = 0.92 and *Q*^2^ = 0.84 indicate that both the model fit and predictive ability meet the high standards for untargeted metabolomics. The results in negative ion mode (Figure 2B) were consistent, with clear separation between the two groups (*R*^2^Y = 0.90, *Q*^2^ = 0.78), further confirming the presence of significant and reproducible differences in metabolic profiles between the two groups. To rule out the risk of overfitting, 200 bootstrap tests were performed on the models for both modes, as shown in Figures 2C, D. The results showed that the *Q*^2^ intercept was −0.43 for the cation mode and −0.45 for the anion mode, both meeting the requirements for the absence of overfitting. This indicates that the PLS-DA model exhibits excellent stability and can be used for subsequent screening of differentially expressed metabolites. Based on this robust model, volcano plots were generated to visualize the distribution of these significant metabolites in both positive (Figure 2E) and negative (Figure 2F) ion modes.

### 3.4 Screening and identification of metabolites with significant differences

**Figure 3:**
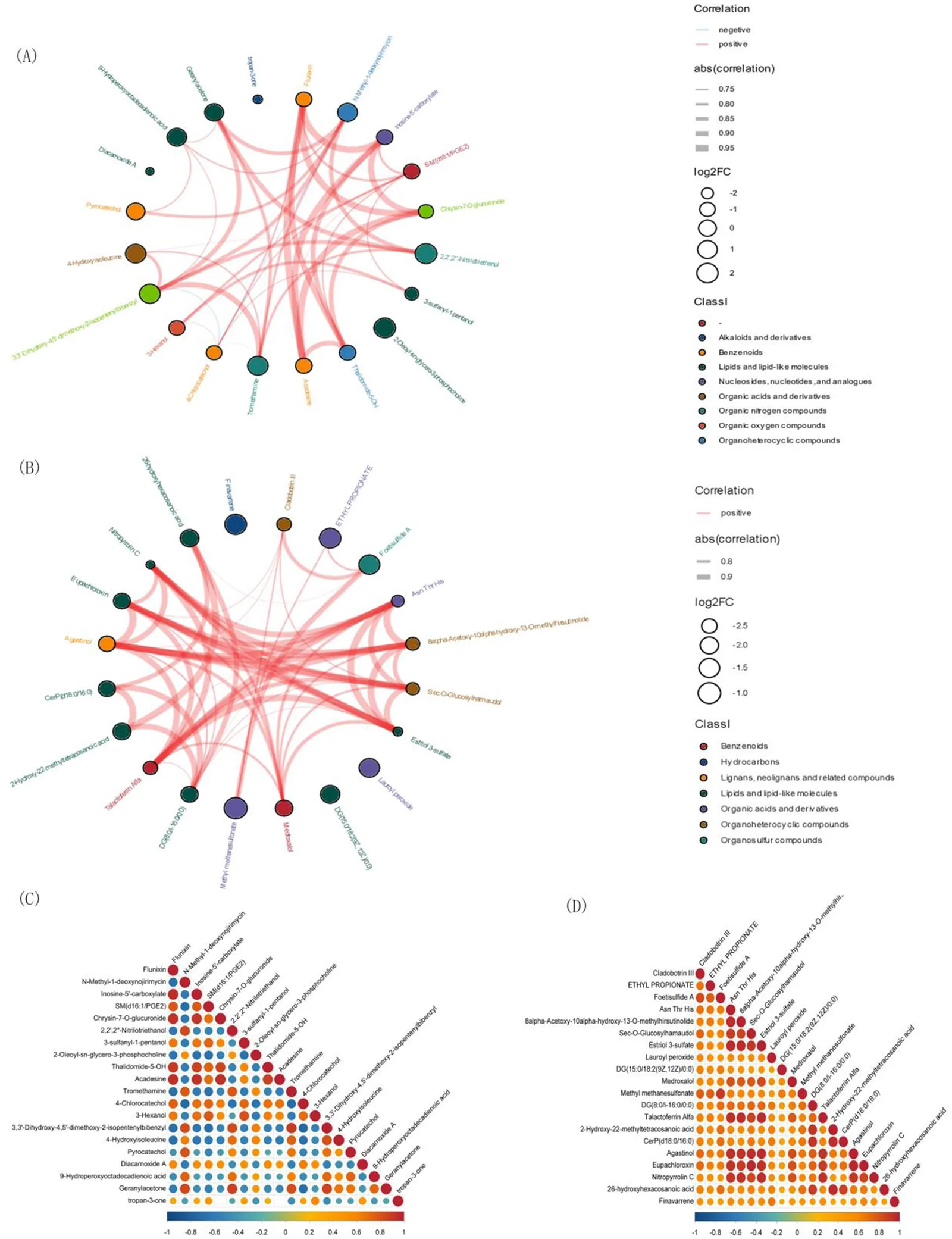
Correlation string plots and Pearson correlation heatmaps of core differentially expressed metabolites in the positive and negative ion modes. **(A, B)** Correlation string plots: Nodes represent differentially expressed metabolites, colored according to chemical class; red lines indicate a significant positive correlation between metabolites; the color of the outer ring around each node indicates the fold change (red for upregulation, blue for downregulation). **(C, D)** Pearson correlation heatmap: displays the strength of correlation (*r*-value) between pairs of core differentially expressed metabolites. Red indicates a positive correlation, blue indicates a negative correlation; the darker the color, the stronger the correlation.

In negative ion mode (Figures 3A, B), compared with the HC group, the ILD group exhibited a total of 367 significantly downregulated metabolites and 109 significantly upregulated metabolites; the number of downregulated metabolites was approximately 3.3 times that of upregulated metabolites, suggesting widespread metabolic depletion or impaired synthesis in the serum of ILD patients. In cation mode, however, a different pattern emerged, with upregulated metabolites (395) slightly outnumbering downregulated metabolites (259), suggesting that the body had activated stress response mechanisms, leading to the accumulation of inflammatory mediators or metabolic intermediates. Pearson correlation analysis (Figures 3C, D) further revealed the interaction patterns among metabolites. in the anion mode (Figure 3D), the vast majority of downregulated metabolites exhibited strong positive correlations (*r* > 0.8), indicating that these lipid and amino acid metabolites are under unified regulation by upstream core pathways; combined with the extensive downregulation observed, this confirms the presence of systemic inhibition of lipid and amino acid metabolism in ILD patients. In cation mode (Figure 3C), significantly positively correlated clusters were also observed among upregulated metabolites, suggesting that specific metabolic pathways are synergistically activated in response to pathological stress; negative correlations between certain metabolite clusters, meanwhile, reflect compensatory regulation between different metabolic modules.

Among the differentially expressed metabolites, DHEA-S exhibited the most significant downregulation trend, being approximately 4.7-fold lower than in the HC group (FC = 0.21, *P* < 0.001). As the most abundant steroid hormone precursor secreted by the adrenal glands, DHEA-S possesses anti-inflammatory and immunomodulatory functions; its significant depletion not only reflects hormonal regulatory imbalance but also suggests the potential disruptive role of the endocrine-immune axis in the pathological progression of ILD, and may be one of the key factors contributing to uncontrolled inflammation and reduced tissue repair capacity.

### 3.5 Functional enrichment of differentially expressed metabolites and metabolic pathway analysis

The above results reveal significant disturbances in the serum metabolome of ILD patients at the level of individual metabolites. To further elucidate the biological functions of these differentially expressed metabolites and the core biochemical pathways in which they are involved, we performed a KEGG pathway enrichment analysis.

**Figure 4:**
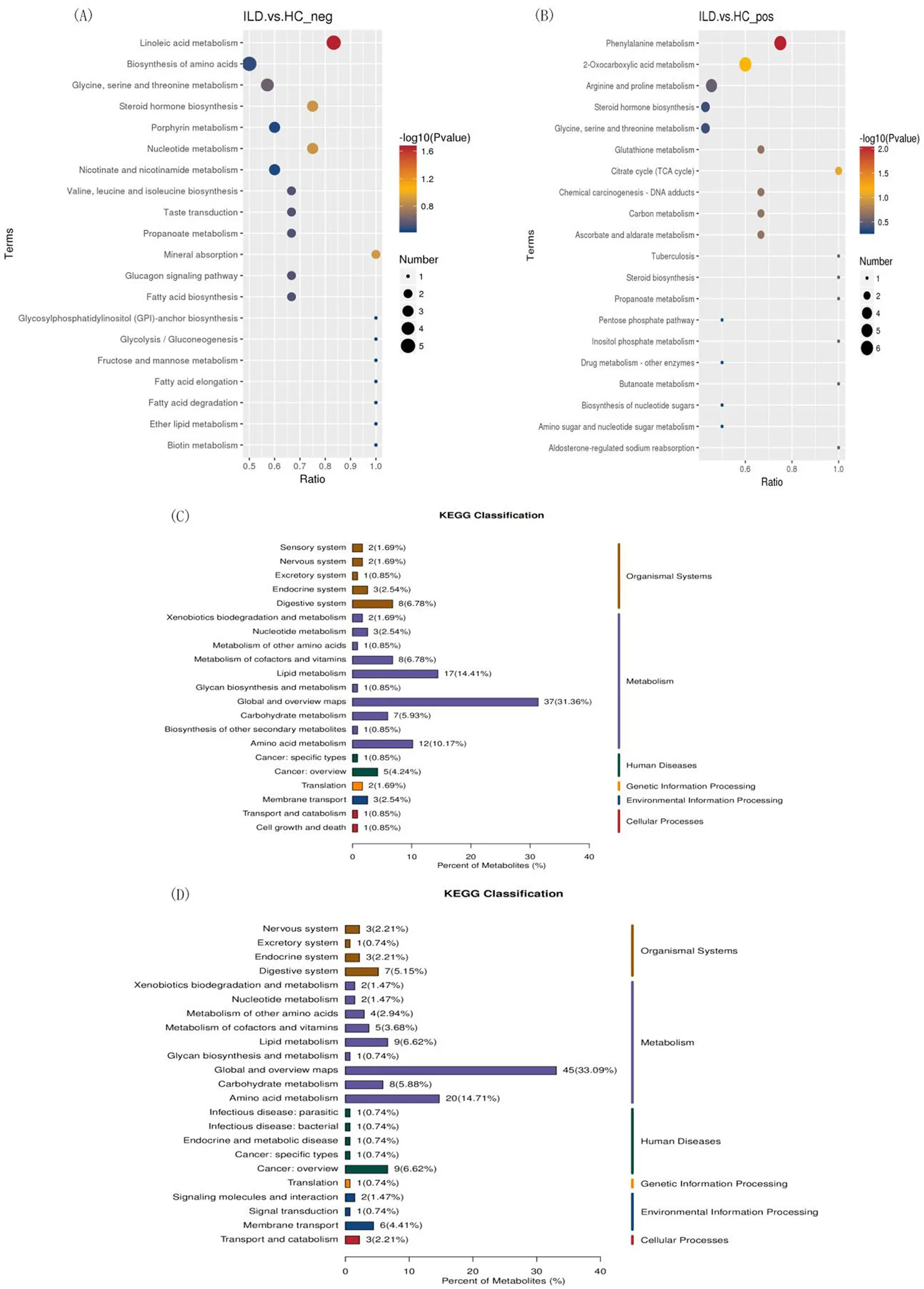
Enrichment and functional annotation of differentially expressed metabolic pathways between the ILD group and the HC group in negative and positive ion modes. **(A, B)** Bubble plots of KEGG pathway enrichment analysis in negative and positive ion modes. Bubble size represents the number of differentially expressed metabolites in the pathway, whilst color indicates enrichment significance [–log10(*P*-value)]; the redder the color, the more significant the result. **(C, D)** KEGG functional category distribution charts for negative ion mode **(C)** and positive ion mode **(D)**, showing the distribution of differentially expressed metabolites across various biological functional categories.

In the anion mode (Figure 4A), differentially expressed metabolites were primarily enriched in pathways such as linoleic acid metabolism, amino acid biosynthesis and phenylalanine metabolism (*P* < 0.05). In the anion mode (Figure 4B), significantly enriched pathways primarily included phenylalanine metabolism, arginine and proline metabolism, and caffeine metabolism. The above results indicate that the pathological process of ILD is accompanied by specific disturbances in amino acid and lipid metabolism. Functional classification analysis further revealed the macro-distribution of metabolic alterations (Figures 4C, D). In the negative ion mode (Figure 4C), differentially expressed metabolites were primarily categorized under lipid metabolism (14.41%), amino acid metabolism (10.17%) and cofactor and vitamin metabolism (6.78%); among these, the “Global and Overview” category accounted for the highest proportion (33.09%), reflecting the widespread involvement of differentially expressed metabolites in multiple fundamental metabolic networks. In the positive ion mode (Figure 4D), amino acid metabolism accounted for the highest proportion (14.71%), followed by the “cancer overview” (6.62%) and lipid metabolism (6.62%). Overall, although the “Global and Overview Profiles” accounted for a high proportion, analysis of specific biochemical pathways revealed that abnormalities in amino acid and lipid metabolism remain the core features of metabolic reprogramming in ILD, suggesting that dysregulation of these two metabolic pathways plays a key role in the pathophysiological process of ILD.

### 3.6 GSEA pathway enrichment analysis

**Figure 5:**
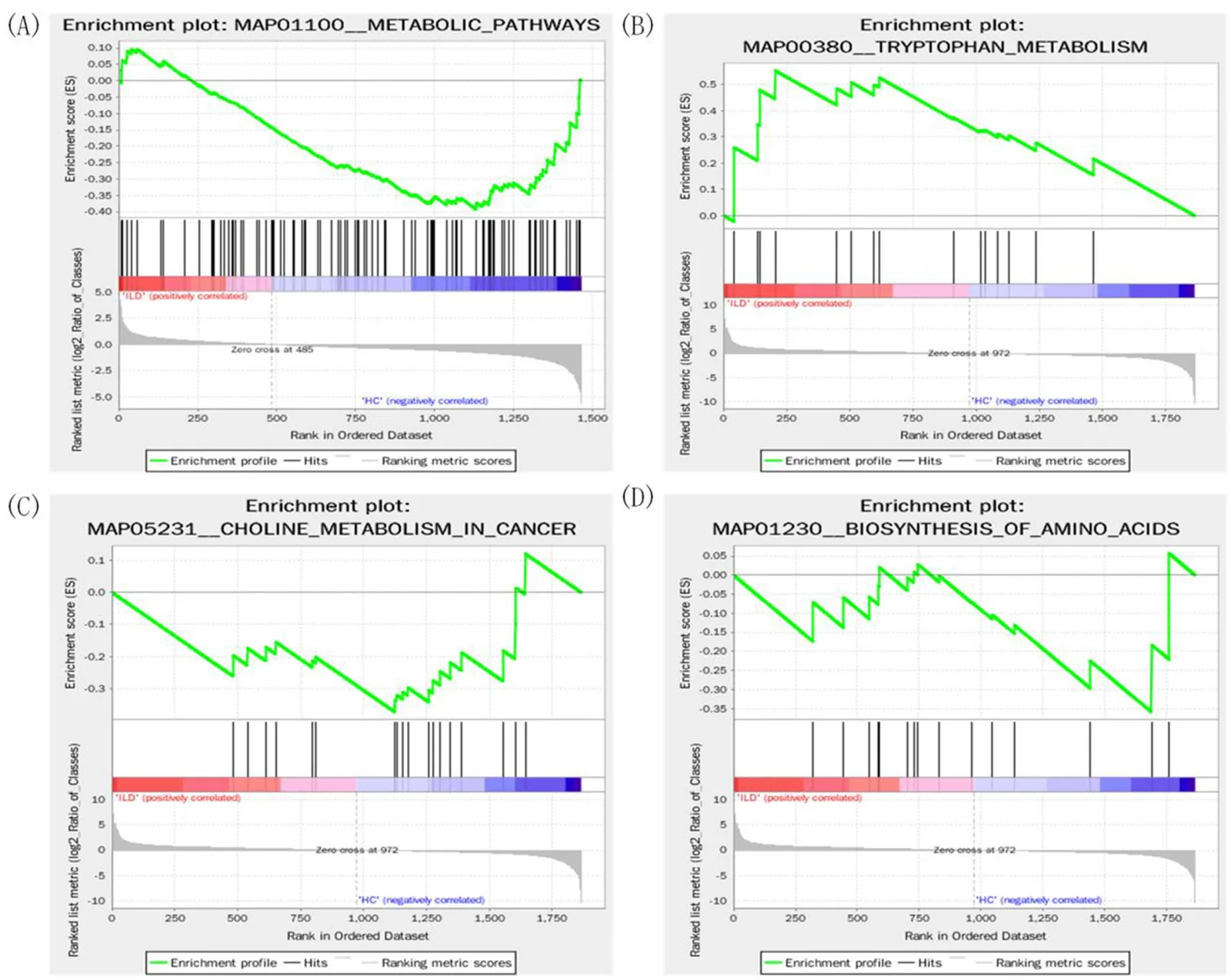
Gene Set Enrichment Analysis (GSEA) of metabolic pathways in the ILD group and the HC group. **(A)** Metabolic pathways in negative ion mode (MAP01100), **(B)** Tryptophan metabolism in positive ion mode (MAP00380), **(C)** Choline metabolism in cancer in positive ion mode (MAP05231), **(D)** Amino acid biosynthesis in positive ion mode (MAP01230).

This study further employed gene set enrichment analysis (GSEA) to comprehensively assess the overall disruption of metabolic pathways. The results in Table 2 show that, although the *p*-values for some pathways did not reach the conventional significance level (*p* < 0.05), the normalized enrichment scores (NES) indicated a clear trend toward metabolic changes. Specifically, as shown in Figure 5, under the negative ion mode, the metabolic pathway (MAP01100) exhibited a negative enrichment trend (NES = −1.258, *P* = 0.094), suggesting that overall metabolic activity in the ILD group may be suppressed. In cation mode, tryptophan metabolism (MAP00380) exhibited the strongest positive enrichment trend (NES = 1.318, *P* = 0.265), indicating that metabolites in this pathway were upregulated in the ILD group, which may be associated with inflammatory activation. Conversely, choline metabolism (MAP05231) and amino acid biosynthesis (MAP01230) exhibited negative enrichment trends (NSE = −0.912, *P* = 0.57; NSE = −0.834, *P* = 0.68), suggesting that lipid remodeling and amino acid synthesis capacity may be downregulated. In summary, the GSEA results reveal the heterogeneity of metabolic changes in patients with ILD, characterized by a general trend toward suppression of metabolic pathways, accompanied by a specific trend toward upregulation of tryptophan metabolism.

**Table 2:** Results of GSEA analysis of metabolic pathways in the ILD group and the HC group.

Ion mode	KEGG ID	KEGG_Term	NES	*P*-value	Direction
Neg	MAP01100	Metabolic pathways	−1.2577772	0.094300516	Down
Pos	MAP00380	Tryptophan metabolism	1.3177913	0.2652024	Up
Pos	MAP02010	ABC transporters	0.8743923	0.6040724	Up^*^
Pos	MAP01230	Biosynthesis of amino acids	−0.8347973	0.6830986	Down^*^
Pos	MAP05231	Choline metabolism in cancer	−0.91231316	0.5745856	Down^*^
Pos	MAP00564	Glycerophospholipid metabolism	−0.5692668	0.96391755	Down^*^
Pos	MAP01100	Metabolic pathways	−0.588251	1	Down^*^

### 3.7 Evaluation of the diagnostic performance of potential metabolic biomarkers

**Figure 6:**
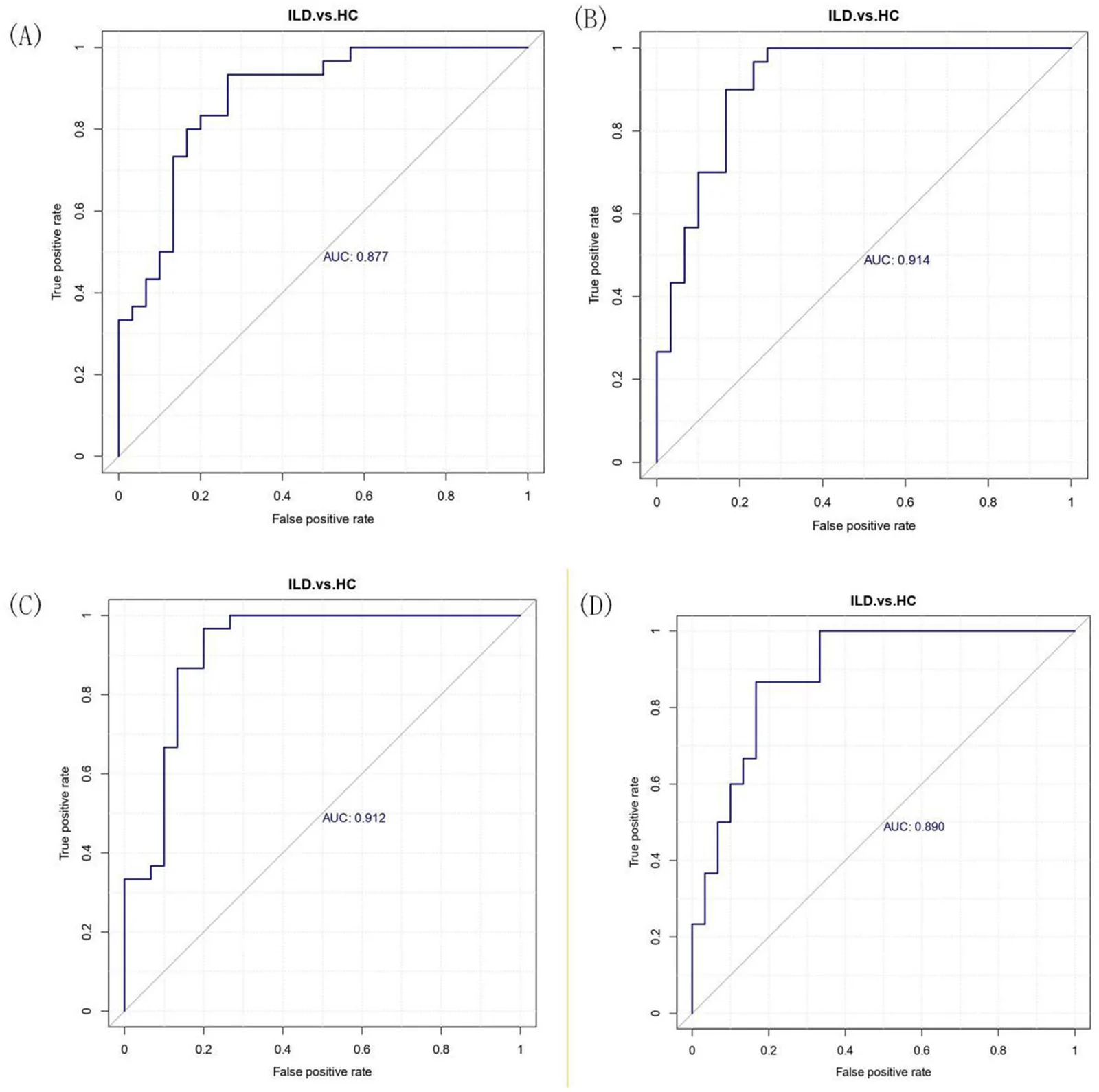
The panels display the receiver operating characteristic (ROC) curves for four specific metabolites: (**A**) DHEA-S (AUC = 0.877); (**B**) DG(15:0/18:2(9Z,12Z)/0:0) (AUC = 0.914); (**C**) Estriol 3-sulfate (AUC = 0.912); and (**D**) Medroxalol (AUC = 0.890). The area under the curve (AUC) is indicated in each plot to demonstrate the discriminative capacity of these candidate biomarkers.

To evaluate the potential of key differential metabolites in distinguishing ILD patients from healthy controls, Receiver Operating Characteristic (ROC) curve analysis was performed (Figure 6). The results indicated that these metabolites exhibited notable discriminative potential within this exploratory cohort.

Specifically, DG(15:0/18:2(9Z,12Z)/0:0), representing linoleic acid metabolism, showed the highest discriminative capacity with an AUC of 0.914. Medroxalol, representing phenylalanine metabolism, followed closely with an AUC of 0.912. Estriol 3-sulfate (representing tryptophan metabolism) and DHEA-S also demonstrated considerable separability, with AUC values of 0.890 and 0.877, respectively. However, it is important to interpret these metrics with caution. Given the limited sample size and the absence of an independent validation cohort, these AUC values should be viewed strictly as preliminary indicators of metabolic separation rather than robust estimates of clinical diagnostic accuracy. Nevertheless, these findings highlight the promising potential of these specific metabolic derivatives as candidate biomarkers for future validation.

## 4 Discussion

Previous studies have widely demonstrated that one of the key pathological features of interstitial lung disease (ILD) is mitochondrial dysfunction, particularly impaired fatty acid β-oxidation (FAO) (17, 18). However, most metabolomics studies have primarily focused on common even-chain fatty acids (such as C16:0 and C18:0), Notably, our study identified a significant elevation in diacylglycerol (DG) containing an odd-chain fatty acid (C15:0). Given that C15:0 is generally not synthesized de novo in the human body and is primarily derived from dietary intake or gut microbiota metabolism (19, 20), this finding suggests that lipid abnormalities in ILD may involve alterations in exogenous metabolic pathways; It is worth noting that this exogenous lipid signal does not exist in isolation, but is specifically incorporated into the molecular structure of diacylglycerol (DG).Diacylglycerol (DG) is not only an intermediate in lipid synthesis but has also been recognized in recent years as a key lipid second messenger capable of promoting fibroblast activation via pathways such as the activation of protein kinase C (PKC) (21). Our study further refines this perspective, revealing that it is the specific DG (15:0/18:2) molecule that is elevated. The existing literature indicates that the composition of fatty acid side chains in lipid molecules (such as C18:2 linoleic acid) directly determines cell membrane fluidity and the release of inflammatory mediators (22–24). Consequently, the accumulation of specific structural DG reflects the specific membrane lipid remodeling that occurs in alveolar epithelial cells in response to injury.

Taking the above points together, we hypothesize that the elevation of DG(15:0/18:2) may reflect two potential pathological mechanisms. Firstly, given that mitochondrial β-oxidation primarily processes even-chain fatty acids, damaged mitochondria may be unable to effectively metabolize odd-chain fatty acids (C15:0), leading to their abnormal retention within the cell in the form of DG. Secondly, although this study—as a cross-sectional observation—cannot establish causality, and serum metabolites can only indirectly reflect the gut microenvironment, the exogenous nature of C15:0 is highly consistent with current hypotheses in the literature regarding the “lung-gut axis.” We hypothesize that specific lipid metabolites derived from the gut microbiota may reach the lungs via the circulatory system and be incorporated into the diacylglycerol (DG) pool in lung tissue; however, this requires further validation in future studies incorporating fecal metagenomics or animal models. Consequently, DG(15:0/18:2) is not only a diagnostic marker but is also likely to be a potential molecular link connecting mitochondrial metabolic defects with gut microbiota dysbiosis.

In classical biochemical pathways, the primary metabolic route for phenylalanine involves hydroxylation to tyrosine, which subsequently serves as a precursor for the synthesis of catecholamine neurotransmitters and melanin (25). However, within the high oxidative stress microenvironment characteristic of ILD, intracellular metabolic homeostasis is often severely disrupted. Our data reveal significant alterations in Medroxalol (a derivative of phenylalanine metabolism), suggesting the activation of a non-classical “shunt” metabolic pathway. Although the exact metabolic pathway of medroxalol remains unclear, based on the context of amino acid metabolism, medroxalol may represent a metabolic adaptive change in chronic inflammation (such as RA-ILD or SSc-ILD). As the existing literature indicates, oxidative stress alters the metabolic flux of amino acids (26, 27). The accumulation of medroxalol may serve as a metabolic fingerprint, marking a decline in the mitochondria's capacity to process aromatic amino acids (28, 29). Secondly, the most compelling mechanism may involve the regulation of TGF-β signaling. According to the latest research findings from 2025 (30), the suppression of TGF-β1 secretion through the regulation of LYVE1+ macrophages is a key mechanism in alleviating pulmonary fibrosis in RA-ILD. As a phenylalanine derivative with a unique structure, medroxalol may be involved in regulating the polarization state of macrophages by influencing intracellular signaling adaptors. We hypothesize that, in patients with ILD, the abnormal accumulation of this metabolite may interfere with the maintenance of the anti-inflammatory phenotype of LYVE1+ macrophages, thereby leading to the dysregulation of the TGF-β1 signaling pathway; however, this requires confirmation through further functional experiments. This view is consistent with the current cutting-edge perspective that regards ILD as a “metabolic-immune” interactive disease.

It is worth noting that, under physiological conditions, sulphation is the primary route of estrogen metabolism, serving to regulate its bioavailability and activity. We hypothesize that the reduction in estriol 3-sulfate may reflect a reprogramming of steroid metabolism in patients with ILD. This metabolic shift may have led to an abnormal accumulation of free estrogen or altered metabolic flux, thereby affecting estrogen receptor (ER) signaling in pulmonary immune cells, such as alveolar macrophages. In light of recent studies highlighting the protective role of LYVE1+ macrophages in pulmonary fibrosis (30), we propose a hypothesis: abnormal metabolism of estriol 3-sulfate may weaken the immune-tolerant environment in the lungs, promoting the polarization of macrophages toward a pro-fibrotic phenotype (M2 type), thereby driving the progression of interstitial lung disease.

In addition to alterations in estrogen metabolism, we have also observed significantly reduced levels of DHEA-S in ILD patients. This phenomenon may reflect a specific manifestation of “endocrine aging” in the course of pulmonary fibrosis. Based on the latest evidence from primate studies in 2024, the adrenal zona reticularis is highly sensitive to aging, a key characteristic of which is the downregulation of LDLR expression with advancing age (31). Given that LDLR serves as the key gateway for the adrenal glands to take up cholesterol for the synthesis of steroid hormones, we hypothesize that the deficiency of DHEA-S in ILD patients is essentially a “secondary synthetic failure” caused by impaired uptake of cholesterol as a raw material. This “synthetic failure” not only cuts off the supply of downstream sex hormones but, more critically, deprives the body of an important endogenous cellular protective mechanism. A groundbreaking structural study published in *Nature Aging* in 2024 revealed that DHEA-S is not merely a hormone precursor, but also an endogenous neurosteroid ligand for the Sigma-1 receptor (32). The study confirmed via crystal structure that DHEA-S binds to the β-barrel domain of the Sigma-1 receptor, acting as a potential receptor agonist involved in regulating endoplasmic reticulum stress (ER stress) and mitochondrial function (32).

Taking these findings together, we position metabolic alterations in DHEA-S as a key upstream hub in the pathogenesis of ILD. Based on the latest research on adrenal aging from 2024 (31), We have proposed a possible pathological model: the “age-like” endocrine dysregulation experienced by patients with ILD leads to impaired DHEA-S synthesis, which in turn weakens the ability of alveolar epithelial cells to respond to endoplasmic reticulum stress due to a deficiency of Sigma-1 receptor ligands (32); Concurrently, the loss of DHEA-S's anti-inflammatory function may trigger abnormally high expression of downstream MMP-7 and MMP-9 (33), thereby compromising the integrity of the pulmonary basement membrane. This persistent matrix degradation and cellular stress ultimately activates FAP+ fibroblasts in the lungs (34), leading to irreversible scarring. We therefore believe that supplementing with DHEA-S or mimicking its signaling pathway that activates the Sigma-1 receptor is a promising avenue for future exploration, with a view to developing potential therapeutic strategies aimed at blocking the release of MMPs and the activation of FAP+ fibroblasts.

Several limitations of this study must be acknowledged. First, the sample size of this single-center cohort was relatively small. Given the high heterogeneity of Interstitial Lung Disease (ILD), such a limited sample size increases the risk of overfitting and potential inflation of AUC values in the diagnostic models. While we identified distinct metabolic signatures, we were unable to perform robust subgroup analyses for specific ILD subtypes (e.g., IPF vs. CTD-ILD) due to the limited number of subjects in each category. Therefore, the current findings should be considered preliminary, and future studies with larger, multi-center, and independent validation cohorts are warranted to confirm the diagnostic value of these metabolites. Second, this study employed a cross-sectional observational design. Although we observed DHEA-S deficiency and elevated odd-chain lipids, and inferred their association with “endocrine aging” and the “gut-lung axis,” these findings represent correlations and do not establish causality. Third, while untargeted metabolomics offers the advantage of broad screening, structural identification of some differential metabolites remains tentative due to database limitations, and further validation using authentic standards via tandem mass spectrometry is required.

## 5 Conclusions

This study utilized untargeted metabolomics to provide a preliminary overview of the serum metabolic profile in interstitial lung disease (ILD), suggesting that the condition is associated with significant systemic metabolic disturbances. The potential metabolic biomarkers identified in the study, along with the “metabolic-immune-endocrine” interaction patterns, provide important clues and hypotheses for the early identification of ILD and future targeted therapies; these findings require further validation through large-scale studies.

## Data Availability

The datasets presented in this study can be found in online repositories. The names of the repository/repositories and accession number(s) can be found in the article/Supplementary material.
